# Effect of selected spices on chemical and sensory markers in fortified rye‐buckwheat cakes

**DOI:** 10.1002/fsn3.329

**Published:** 2016-01-15

**Authors:** Małgorzata Przygodzka, Henryk Zieliński, Zuzana Ciesarová, Kristina Kukurová, Grzegorz Lamparski

**Affiliations:** ^1^ Division of Food ScienceInstitute of Animal Reproduction and Food Research of the Polish Academy of SciencesTuwima 10P.O. Box 5510‐748Olsztyn 5Poland; ^2^ National Agriculture and Food Centre – Food Research InstitutePriemyselná 4P.O. Box 25824 75Bratislava 26Slovak Republic

**Keywords:** Antioxidant activity, buckwheat flour, cakes, Maillard reaction, sensory evaluation, spices

## Abstract

The aim of this study was to find out the effect of selected spices on chemical and sensorial markers in cakes formulated on rye and light buckwheat flour fortified with spices. Among collection of spices, rye‐buckwheat cakes fortified individually with cloves, nutmeg, allspice, cinnamon, vanilla, and spice mix revealed the highest sensory characteristics and overall quality. Cakes fortified with cloves, allspice, and spice mix showed the highest antioxidant capacity, total phenolics, rutin, and almost threefold higher available lysine contents. The reduced furosine content as well as free and total fluorescent intermediatory compounds were observed as compared to nonfortified cakes. The FAST index was significantly lowered in all cakes enriched with spices, especially with cloves, allspice, and mix. In contrast, browning index increased in compare to cakes without spices. It can be suggested that clove, allspice, vanilla, and spice mix should be used for production of safety and good quality cakes.

## Introduction

Spices have been used by human since ancient times. According to the U.S. Food and Drug Administration (US FDA) spice is an “aromatic vegetable substance in the whole, broken, or ground form, the significant function of which in food is seasoning rather than nutrition” and from which “no portion of any volatile oil or other flavoring principle has been removed” (Sung et al. 2012). The compilation of current trends in bakery technology to enhance antioxidant activity of bakery products was widely described by Dziki et al. (2014). At the top of the list, spices have been suggested as a well‐recognized source of compounds with antioxidant potential (Hinneburg et al. 2006; Wojdyło et al. 2007; Charles 2013). Recently, the inquisitive studies on antioxidant capacities of spices employing updated analytical methods were reported by Przygodzka et al. (2014). According to data collected in Food Frequency Questionnaire, the average of spices/herbs intake was estimated as 1.1 grams per day for one person what revealed that spices are important contributor of antioxidants to our diet (Carlsen et al. 2011). Spices are mainly employed as flavoring and color agents, whereas potential use to preservation food and disease prevention has been already studied (Kaefer and Milner 2008; Cazzola and Cestaro 2014; Embuscado 2015). Spice application was demonstrated by Illupapalayam et al. (2014). Their probiotic‐yogurt with cardamom, cinnamon, and nutmeg has increased sensorial acceptability among consumers, besides spice addition increased overall antioxidant activity of this functional product.

Presently, consumers seeking for new food products are focused on joining two aspects: a taste and functional properties (Wójtowicz et al. 2013). The functional properties of innovative products in prevention or therapy support in selected diseases are desirable. Anticancer, antiallergic, antiviral, cholesterol‐reducing, blood pressure‐reducing, and arteriosclerosis‐reducing were ascribed as buckwheat's healing effects (Krkošková and Mrázová 2005). In this trend, buckwheat‐based product with spices addition can be a good alternative to inclusion in varied and balanced diet. Moreover, several studies approved consumer acceptability of buckwheat‐based products (Wronkowska et al. 2008; Filipčev et al. 2011; Sedej et al. 2011; Chlopicka et al. 2012). The high sensorial acceptability of 30% buckwheat flour incorporation in baked products was reached.

In this study, the recipe of rye‐buckwheat cakes (RBC) was enriched with one spice form the list including: anise, allspice, cardamom, cinnamon, cloves, coriander, fennel, ginger, nutmeg, star anise, vanilla, white pepper, and commercial spice mix for ginger cakes. The sensory evaluation of cakes was used as a tool for selection cakes accepted by sensory panel. It seems to be rationale to use Maillard reaction (MR) products as markers for description quality of RBC fortified with spices. It is well‐known that MR products are responsible for the development of color, taste, and aroma as well as the nutrients loss of thermally treated food (Markowicz Bastos and Gugliucci 2015). Virág et al. (2013) stated that remaining lysine after baking process is a good indicator of the progress of MR and important to monitor its content as essential amino acids. Several unfavorable food contaminants are simultaneously formed in thermal processing. During early step of MR, the nutritionally valued available lysine can be converted into furosine, a heat‐treatment marker (Gökmen et al. 2008; Giannetti et al. 2014). The advanced stage of MR is characterized by the formation of fluorescence compounds with regard to advanced glycation end‐products formation and monitoring protein degradation by FAST index (Delgado‐Andrade et al. 2007; Liogier de Sereys et al. 2014). Positively, melanoidins formed in the final stage of MR are responsible for the color formation and possess the ability to scavenge free radicals (Langner and Rzeski 2014). It was concerned that MR products formation in a model systems and food products can be reduced/increased by an application of substances having a high antioxidant potential (Marková et al. 2012; Oral et al. 2014; Cheng et al. 2015).

The aim of this study was to find out an impact of selected spices on Maillard reaction progress and sensory quality of RBC fortified with spices. Therefore, analysis of selected chemical and sensorial markers such as quercetin 3‐rhamnosylglucoside (rutin)—the main buckwheat flavonoid, available lysine, total phenolics contents (TPC), antioxidant capacity (AC) of cakes using extracts scavenging activity against ABTS^•+^ radical cation and against superoxide anion radicals (O_2_^•−^) measured by the photochemiluminescence method (PCL), and furosine, fluorescent compounds, and melanoidins, were addressed in this study. To determine the impact of thermal treatment on protein damage, FAST index was calculated.

## Materials and Methods

### Chemicals and reagents

2,2′‐Azinobis(3‐ethylbenzothiazoline‐6‐sulphonic acid) diammonium salt (ABTS), 6‐hydroxy‐2,5,7,8‐tetramethylchroman‐2‐carboxylic acid (Trolox), rutin (quercetin‐3‐rutinoside), lysine (N^*α*^‐acetyl‐L‐lysine), and pronase E (*Streptomyces griseus lyoph.)* were purchased from Sigma (Sigma Chemical Co., St. Louis, MO). PCL ACW (Antioxidant Capacity of Water‐soluble substances) kit for PCL assay was from Analytik Jena AG (Jena, Germany). o‐phtaldialdehyde for fluorescence (OPA) and sodium dodecylsulfonate (SDS) were supplied by Fluka (Buchs, Switzerland). Furosine (2‐furoylmethyl‐lysine) was purchased from PolyPeptide (Strasbourg, France). Acetonitrile and methanol (HPLC purity) were provided by POCh (Gliwice, Poland). Water was purified with Mili‐Q‐system (Millipore, Bedford, MA).

### Formulation of rye‐buckwheat ginger cakes enhanced with spices

The cakes were baked using rye flour blended with light buckwheat flour in ratio 70:30 (w/w). The making process involved dough preparation by mixing flours, honey, and sugar. Each one of selected spices (2% on flour mixture basis; w/w) from the list: anise, allspice, cardamom, cinnamon, cloves, coriander, fennel, ginger, nutmeg, star anise, vanilla, white pepper, and commercial spice mix for ginger cake, was used in RBC recipe. According to the producer's declaration, commercial spice mix contained cinnamon, pepper, clove, anise, coriander, fennel, and nutmeg. The amounts of ingredients added to make each type of cake are presented in Table 1. The dough was cut into 0.5‐cm‐thick disks of 5.5 cm diameter and baked at 180°C for 18 min in a DC‐32E electric oven (Sveba‐Dahlen, Fristad, Sweden). Finally, the cakes were freeze‐dried and grounded into powder. The powdered samples were sieved through a 60‐mesh screen and then stored at −20°C until analyzed.

**Table 1:** Formula of rye‐buckwheat cakes fortified with selected spices: anise, allspice, cardamom, cinnamon, cloves, coriander, fennel, ginger, nutmeg, star anise, vanilla, white pepper, and commercial mix of spices for ginger cake

Ingredients	Control cake	Rye‐buckwheat cake with spice addition
Rye flour (T‐720) (g)	70	70
Light buckwheat flour (g)	30	30
Buckwheat honey (g)	50	50
Sugar (g)	20	20
Baking powder (g)	3	3
Butter (g)	25	25
Selected spice (g)	0	2

### Sensory evaluation

Twenty‐four attributes related to the appearance, odor, taste, and texture of rye‐buckwheat ginger cakes with spices were selected and thoroughly used to during profiling procedure. Sensory characteristics and overall quality of ginger cakes were evaluated according to international unified standards (ISO/DIS 1998). A six‐member trained panel judged ginger cakes in a 10‐point scale (0—for weak, 10—for very good) using quantitative descriptive analysis to determine differences between each type of ginger cakes (Stone et al. 2012). The description of sample preparation and standardized procedure of sensory evaluation were in details presented by Zieliński et al. (2012).

Overall acceptability of each sample was evaluated in relation to the sensory preferences on the basis of overall appearance, aroma, taste, and texture, in a 10‐point hedonic scale, where: not accept, and 10 fully accept. The profiling analysis of all samples was run in duplicate (two series) proceeded by introduction session. Ginger cakes were considered as acceptable if their mean scores for overall acceptability were above 6 (Kowalska et al. 2012).

### Preparation of extracts from RBC

Rye‐buckwheat cake powders (100 mg) were extracted with 1 mL of 65% (v/v) ethanol. After ultrasonic vibration for 30 sec, the solution was mixed and centrifuged for 5 min at 5000× g at 4°C. That step was repeated five times and the supernatants were collected into 5‐mL flask. Final extracts concentration was 20 mg/mL. Ethanol extracts were prepared in triplicate. Next extracts were stored at −20°C until analysis of rutin content, total phenolic compounds (TPC), and AC by ABTS and PCL ACW assays.

### Determination of total phenolic content (TPC) and rutin

The TPC was determined with Folic‐Ciocalteu reagent as it was described in details by Przygodzka et al. (2014). TPC was standardized against gallic acid and expressed in terms of mg gallic acid equivalents (GAE)/g dry matter. The content of rutin in ginger cakes was determined with HPLC (Shimadzu, Japan) with UV detector (SPD‐10A) set up 330 nm as it was recently described by Zielińska et al. (2010). For quantitative analysis, rutin standard was prepared in triplicate at five concentrations within the range 1.0–40 *μ*M. All solutions were filtered through a 0.45 *μ*m nylon membrane before use. The results were expressed in *μ*g per g of dry matter.

### Antioxidant capacity determination

The AC of RBC enhanced with spices was determined by ABTS and photochemiluminescence (PCL ACW) assays as it was described in details by Przygodzka et al. (2014). The results provided by ABTS and PCL ACW methods were expressed as *μ*mol of Trolox equivalents (TE)/g DM.

### Available lysine content determination

The OPA assay as described by Michalska et al. (2008) was employed to determine available lysine content using the microplate reader (Infinite^®^ M1000 PRO, Tecan, Switzerland). Exactly 50 *μ*L of sample, 100 *μ*L of OPA reagent, and 100 *μ*L of water were added to well and incubated for 3 min (96‐well microplate; Porvair Sciences, Norfolk, UK). Then the fluorescence reading was measured at extinction wavelength 340 nm and emission wavelength 455 nm. Quantitative analysis was performed by the external standard method, employing a calibration curve of N^*α*^‐acetyl‐L‐lysine ranged from 10 to 250 *μ*M. Each result is a mean of three independent extractions.

### Maillard reaction products determination

#### Furosine content determination

According to Delgado‐Andrade et al. (2007), 30 mg of cake sample was hydrolyzed with 4 mL of 4.9 M HCl at 110°C for 23 h in a Pyrex screw‐capped vial with PTFE‐faced septa. Hydrolysis tubes must be sealed under nitrogen. After that the hydrolysates was centrifuged for 10 min. A 0.5 mL portion of the supernatant was applied to a Sep‐pak C18 cartrigde (Millipore) conditioned with 5 mL of methanol and 10 mL of distilled water, then eluted with 3 mL of 3M HCL and evaporated under vacuum. The dried sample was dissolved in 1 mL of a mixture of water, acetonitrile, and formic acid (95:5:0.2) before HPLC analysis.

The furosine was quantified by HPLC system (Shimadzu, Japan) comprised of a controller (SCL‐10AVP), a PDA detector (SPD‐M10AVP). A Cadenza CD‐C18 column (250 × 2 mm, 3 *μ*m, Imtakt, Kyoto, Japan) at 35°C. The mobile phase consisted of a solution of 5 mM sodium heptanases sulfonate containing 20% of acetonitrile and 0.2% of formic acid. The elution was isocratic and the flow rate was 0.2 mL/min. The UV detector was set at 280 nm. Calibration curve was made by the external standard of furosine 0.2–9 *μ*g/mL.

### Measurement of MR fluorescence intermediatory compounds and FAST index calculation

The fluorescence of free, linked‐to‐protein, and total intermediary compounds (FIC) was determined after sample extraction and further enzymatic hydrolysis using pronase E according to Delgado‐Andrade et al. (2006). Readings were recorded in a luminescent spectrofluorimeter (LS 50B; Perkin Elmer, Waltham, USA) setting at *λ*_ext._ = 347 nm and *λ*_em._ = 415 nm. Tryptophan fluorescence Trp_FL_ was measured at *λ*_ext._ = 290 nm and *λ*_em._ = 340 nm. Results are expressed in fluorescence intensity (FI) per mg of sample DM. The FAST index was calculated as recently reported by Zieliński et al. (2012) with a one novelty modification based on the use of fluorescent compounds linked‐to‐proteins for index calculation. The samples were analyzed in triplicate and FAST index data were expressed as a percentage (%).

### Brown pigments assay

Formation of brown pigments was estimated as reported in details by Zieliński et al. (2012). All measurements were performed in triplicate. Results were expressed as arbitrary absorbance units.

### Statistical analysis

The results of the chemical analyses are given as the means and the standard deviation of three independent measurements. Statistical one‐way analysis of variance (ANOVA) using Fischer test was performed. The significance level was set at *P* < 0.05. The correlation test between rutin content, antioxidant ability, and MRPs formation was performed and the Pearson correlation coefficients were calculated. Statistical analyses were performed using software package (StatSoft Inc., v. 7.1, Tulsa, OK).

## Results and Discussion

### Consumer acceptance

**Figure 1:**
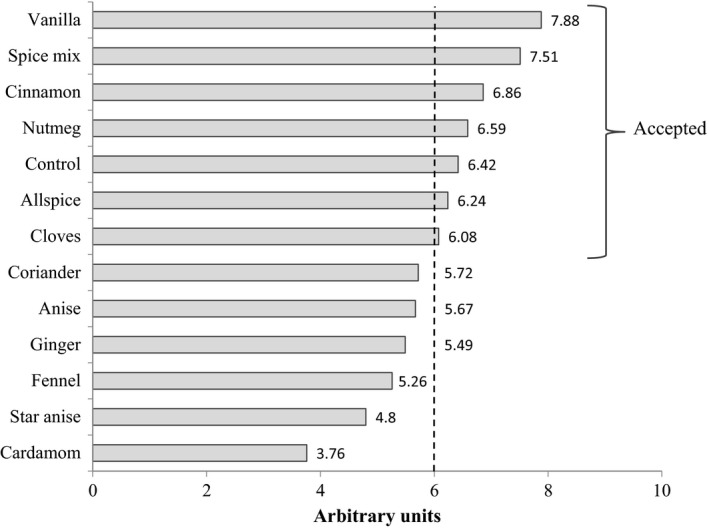
The overall quality of rye‐buckwheat cakes fortified with spices addition.

The overall quality of RBC made of light buckwheat flour encorporated with selected spices is presented on Figure 1. The overall acceptability for control cake was 6.4, in comparison its sensorial score was higher than for cakes made of buckwheat and wheat flour proposed by Kaur et al. (2014). The RBC fortified with spices showed following rank of acceptability: cakes with vanilla (7.9), with spice mix (7.5), with cinnamon (6.9), and with nutmeg (6.6). Also high acceptability showed cakes fortified with allspice (6.2) and cloves (6.1). Taking into account the overall acceptability rating, it was decided to use RBC fortified with allspice, cinnamon, cloves, spice mix, nutmeg, and vanilla for further chemical analysis.

### Sensory evaluation

**Figure 2:**
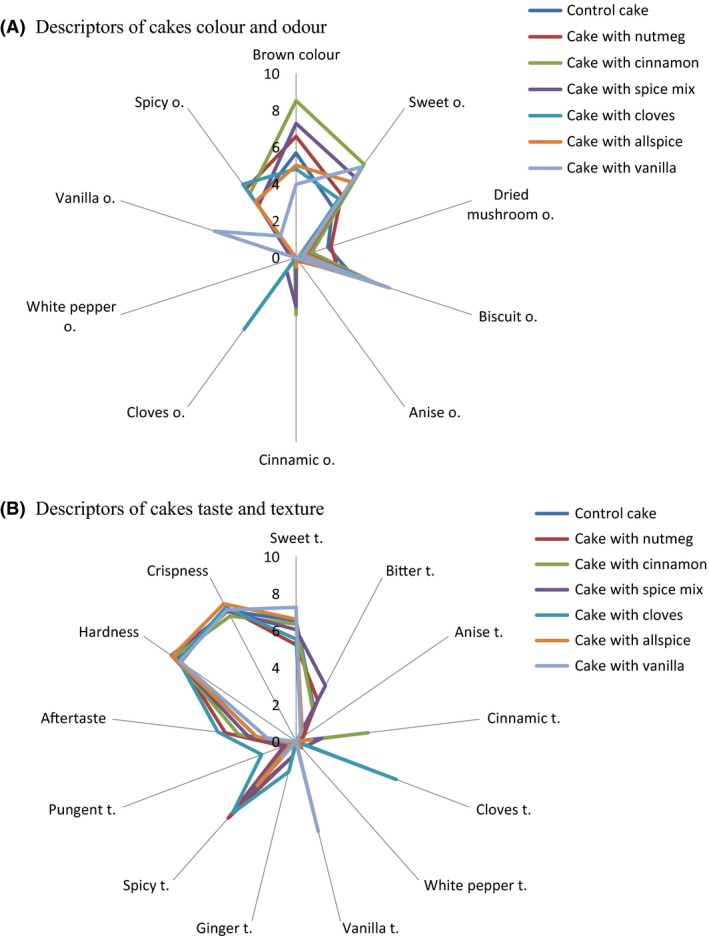
Sensory profiles of rye‐buckwheat cake without spice (control cake) and rye‐buckwheat cakes fortified with selected spices: nutmeg, cinnamon, spice mix, cloves, allspice, and vanilla. O, attributes of odor; t, attributes of taste.

In order to observe the above differences in the analyzed samples more clearly, the sensory profiles of RBC as control and cakes enriched with clove, cinnamon, allspice, nutmeg, vanilla, and spice mix were displayed as spider diagrams in Figure 2A,B. The mean sensory ratings for the samples and the analysis of variance are presented in Table 2. The buckwheat honey, sugar as well as cinnamon and vanilla usage can contribute on high level of sweet taste and odor in presented cakes. The sweetness can be mitigated bitter taste and aftertaste of buckwheat flour. According to Pauly et al. (2013), the high values of hardness can be linked to type of buckwheat flours used in recipe. ANOVA showed that there were significant differences in the intensity of attributes such as brown color, odor descriptors: “sweet,” “biscuit,” “cinnamic,” “cloves,” “vanilla,” and “spicy”; taste descriptors: bitter, “cinnamic,” “cloves,” “vanilla,” “spicy,” pungent, and aftertaste. The high score of acceptability for cake with vanilla was involved with significantly low contribution of negative attributes as spicy, pungent taste, and aftertaste, masked by intensive biscuit and vanilla odor, and vanilla taste. The highest scores of cinnamic odor and cinnamic taste, vanilla odor and vanilla taste as well as cloves odor and cloves taste were characteristic for cakes with cinnamon, vanilla, and cloves, respectively. It can be concluded that 2% of spice addition was sufficiently for differentiation between samples. Therefore, the sensory evaluation proved that addition of spices to RBC formulation increased the sensory quality of products.

**Table 2:** Descriptive analysis of results based on the analysis of variance (ANOVA) performed on rye‐buckwheat cakes (RBC) fortified with selected spices

Attribute		Rye‐buckwheat cakes						
		Control	Cloves	Nutmeg	Allspice	Cinnamon	Vanilla	Spice mix
1	Brown color	5.67^cd^	4.81^de^	6.58^bc^	5.01^cde^	8.51^a^	3.98^e^	7.28^ab^
2	Sweet o.	3.38^c^	3.93^bc^	4.21^bc^	5.08^ab^	6.26^a^	6.08^a^	5.41^ab^
3	Dried mushroom o.	1.82^a^	0.25^a^	1.99^a^	0.54^a^	0.89^a^	0.36^a^	0.60^a^
4	Biscuit o.	3.23^bc^	0.97^d^	2.42^cd^	3.95^abc^	4.67^ab^	5.32^a^	4.31^ab^
5	Anise o.	0.01^a^	0.01^a^	0.20^a^	0.14^a^	0.03^a^	0.01^a^	0.02^a^
6	Cinnamic o.	0.09^b^	0.01^b^	0.00^b^	0.51^b^	3.10^a^	0.02^b^	2.66^a^
7	Cloves o.	0.00^c^	4.81^a^	0.02^c^	0.00^c^	0.13^c^	0.01^c^	0.88^b^
8	White pepper o.	0.07^a^	0.01^a^	0.01^a^	0.02^a^	0.02^a^	0.02^a^	0.02^a^
9	Vanilla o.	0.09^c^	0.01^c^	0.08^c^	0.02^c^	0.12^c^	4.66^a^	0.34^b^
10	Spicy o.	3.61^a^	4.92^a^	4.62^a^	3.76^a^	4.27^a^	1.45^b^	3.48^a^
11	Sweet t.	6.53^a^	5.53^a^	5.23^a^	6.61^a^	6.35^a^	7.25^a^	6.04^a^
12	Bitter t.	2.06^ab^	0.60^b^	2.51^a^	0.70^b^	1.95^ab^	0.51^b^	3.41^a^
13	Anise t.	0.51^a^	0.02^a^	0.46^a^	0.02^a^	0.02^a^	0.02^a^	0.02^a^
14	Cinnamic t.	0.02^b^	0.02^b^	0.02^b^	0.85^b^	3.92^a^	0.02^b^	1.40^b^
15	Cloves t.	0.02^c^	5.79^a^	0.02^c^	0.02^c^	0.00^c^	0.01^c^	0.62^b^
16	White pepper t.	0.01^a^	0.02^a^	0.44^a^	0.38^a^	0.02^a^	0.02^a^	0.02^a^
17	Vanilla t.	0.34^b^	0.03^b^	0.01^b^	0.02^b^	0.02^b^	5.01^a^	0.06^b^
18	Ginger t.	0.01^a^	1.68^a^	0.83^a^	0.02^a^	0.01^a^	0.03^a^	0.84^a^
19	Spicy t.	4.68^ab^	5.17^ab^	5.54^a^	3.18^c^	4.06^bc^	1.13^d^	4.08^bc^
20	Pungent t.	0.13^b^	1.99^a^	0.72^b^	0.26^b^	0.30^b^	0.02^b^	0.61^b^
21	Aftertaste	2.15^bc^	4.26^a^	3.85^ab^	2.17^bc^	3.13^abc^	1.57^c^	2.64^abc^
22	Hardness	7.98^a^	8.19^a^	8.19^a^	8.41^a^	7.66^a^	8.03^a^	7.96^a^
23	Crispness	6.42^a^	6.08^a^	6.59^a^	6.24^a^	6.86^a^	7.88^a^	7.51^a^

The cakes were marked in a 10‐point scale (0—for weak, 10—for very good). Means in each row with the same letters do not have significant differences (Fisher test, *P* < 0.05). o, attributes of odor; t, attributes of taste.

### The total phenolic content (TPC) and rutin determination

The content of rutin and total phenolic compounds in RBC fortified with selected spices is compiled in Table 3. The addition of spices to RBC formula resulted in increase in TPC in comparison to control cake. The significantly highest TPC values in cakes with spice mix, cinnamon and cloves were observed, 4.84‐fold, 2.03‐fold, and 1.97‐fold, respectively (*P* < 0.05). Our results are in accordance to Przygodzka et al. (2014) who noted high TPC values for spice mix, cloves, and cinnamon. The level of rutin was significantly higher in cakes after cloves and allspice application in comparison to control cake (3.07 and 1.66 times, respectively). The novel RBC possess rutin content 5.5 times and 6 times higher than in gluten‐free rice‐light buckwheat and rice‐wholegrain buckwheat (70:30, w/w) breads, respectively (Sakač et al. 2011), which might be related to differentiations of rutin content in buckwheat flours and application of buckwheat honey in the cakes recipe. Additionally, our TPC results for control cake are in agreement with the results of these gluten‐free breads (Sakač et al. 2011).

**Table 3:** The content of rutin, total phenolic compounds, and antioxidant capacity of rye‐buckwheat cakes enhanced with selected spices

Type of cake	Rutin (*μ*g/g DM)	TPC (mg GAE/g DM)	Antioxidant capacity (*μ*mol TE/g DM)	
			PCL ACW	ABTS
Rye‐buckwheat control cake	104.36 ± 3.75^cd^	1.12 ± 0.03^g^	6.15 ± 0.51^d^	21.13 ± 0.88^f^
Rye‐buckwheat cake with vanilla	100.63 ± 2.33^d^	1.32 ± 0.08^f^	5.17 ± 0.83^d^	21.87 ± 1.21^f^
Rye‐buckwheat cake with cinnamon	101.62 ± 6.17^d^	2.28 ± 0.05^b^	8.69 ± 0.87^c^	49.38 ± 0.19^c^
Rye‐buckwheat cake with cloves	319.80 ± 3.51^a^	2.11 ± 0.04^c^	23.30 ± 1.00^b^	55.52 ± 2.73^b^
Rye‐buckwheat cake with allspice	173.19 ± 7.52^b^	1.84 ± 0.17^d^	9.25 ± 0.22^c^	40.86 ± 2.28^d^
Rye‐buckwheat cake with nutmeg	100.84 ± 3.53^d^	1.56 ± 0.10^e^	5.78 ± 0.13^d^	30.49 ± 0.84^e^
Rye‐buckwheat cake with spice mix	111.16 ± 3.50^c^	2.70 ± 0.09^a^	31.56 ± 0.05^a^	63.24 ± 1.31^a^

TPC (total phenolic content) is expressed in mg of gallic acid equivalents/g of dry matter (mg GAE/g DM). Antioxidant capacity measured by ABTS and PCL ACW methods is expressed in *μ*mol of Trolox equivalents (TE)/g DM. Values are means ± standard deviation (*n *=* *3). Values in each column with different small superscript letters are significantly different (Fisher test, *P *<* *0.05). DM, dry matter.

A weak correlation between rutin and TPC was noted (*r* = 0.23). It can be suggested that other flavonoid compounds extracted from spices have higher contribution on the antioxidant properties of RBC. Moreover, the negative correlation was found between TPC and bitter taste (*r* = −0.61). It can be said that phenolic compounds increased bitterness, our findings are in accordance to information collected by Shahidi and Naczk (1995).

### Antioxidant properties

The 2% spices substitution in the formulation of cakes made of rye and light buckwheat flour resulted in significant differences (*P *<* *0.05) in the AC determined against scavenging ability of ABTS^•+^ and O_2_^−•^ (PCL ACW method) radicals. The results for AC determination are presented in Table 3. Sorted by AC measured by ABTS method RBC supplemented with spice mix has the highest antioxidant value followed by cloves, cinnamon, then allspice, nutmeg and finally vanilla cakes. Significantly highest results were obtained for RBC with spice mix, cloves, cinnamon and allspice, 2.99, 2.63, 1.93 –times higher than in control cake. The antioxidant potential evaluation by PCL ACW method for RBC was listed as follows: spice mix> cloves> allspice ≈ cinnamon> nutmeg≈ vanilla. The addition of spice mix and cloves was more effective in enhancing antioxidant activity, as evaluated by means of PCL ACW, which increased 6.10‐fold and 4.50‐fold, respectively. These results are in agreement with findings of Hossain et al. (2008), which indicated that cloves and cinnamon have the highest AC among other spices.

Moreover, the TPC and rutin contribution on AC overall was expressed by correlation coefficient. The strong correlation between TPC/ABTS and TPC/PCL ACW data (*r* = 0.97 and *r* = 0.81, respectively) were observed. According to studies of Bi et al. (2015), the strong correlation between TPC and AC measured by ABTS was observed for cloves extracts. It may suggest that active compounds from cloves have high contribution to antioxidant overall capacity of RBC. However, the weaker correlations for rutin versus PCL (*r* = 0.43) and rutin versus ABTS (*r* = 0.44) were noted.

### Available lysine determination

The results for available lysine amount after thermal processing are shown in Table 4. Available lysine values of 0.52 mg/g DM was found in control RBC without condiments supplementation. According to the obtained results for available lysine in rye‐buckwheat cake with condiments addition a protective effect on lysine blockage was found. The statistically significantly most high lysine blockage content in cloves, allspice, and spice mix was noted (2.75, 2.64, 2.20 times higher). The observation of protective effect of spices on lysine blockage in cakes was confirmed by positive correlation between OPA values and rutin and TPC contents (*r* = 0.74 and 0.63, respectively), as well as AC measured by ABTS (*r* = 0.74) and PCL ACW (*r* = 0.62). On the basis of these results, it can be concluded that spices positively influenced the baking process and increased the nutritional value of the product.

**Table 4:** Data on Maillard reaction products in rye‐buckwheat cakes fortified with spices

Type of cake fortified with spices	Available lysine (mg/g DM)	Furosine (*μ*g/g)	Free FIC (FI/mg DM)	Total FIC (FI/mg DM)	Linked‐to‐protein (FI/mg DM)	Tryptophan (FI/mg DM)	FAST (%)	Browning (AU)
Control	0.59 ± 0.04^d^	510.8 ± 12.0^a^	77.44 ± 1.44^d^	166.57 ± 2.20^c^	89.13 ± 1.76^bc^	19.30 ± 1.00^cd^	462 ± 8^b^	0.36 ± 0.02^e^
Vanilla	0.67 ± 0.09^d^	488.4 ± 6.5^b^	116.14 ± 0.97^a^	201.68 ± 6.12^a^	85.54 ± 5.15^c^	17.68 ± 0.73^d^	484 ± 2^a^	0.41 ± 0.01^d^
Cinnamon	0.95 ± 0.03^c^	450.2 ± 9.7^c^	69.06 ± 3.35^b^	182.60 ± 4.18^b^	113.54 ± 7.53^a^	30.87 ± 0.81^b^	368 ± 4^e^	0.49 ± 0.01^c^
Cloves	1.62 ± 0.06^a^	111.7 ± 7.2^f^	69.21 ± 1.04^b^	142.36 ± 6.23^e^	73.15 ± 7.27^d^	21.92 ± 1.21^c^	230 ± 9 ^g^	0.68 ± 0.01^a^
Allspice	1.56 ± 0.07^a^	292.9 ± 5.6^d^	65.63 ± 1.65^c^	153.26 ± 3.76^d^	87.63 ± 5.46^bc^	19.44 ± 0.22^cd^	451 ± 3^c^	0.52 ± 0.01^b^
Nutmeg	0.99 ± 0.02^c^	458.6 ± 10.8^c^	114.91 ± 1.11^a^	200.92 ± 8.34^a^	86.01 ± 7.23^c^	31.87 ± 3.61^b^	392 ± 4^d^	0.48 ± 0.02^c^
Spice mix	1.30 ± 0.16^b^	220.5 ± 10.1^e^	54.02 ± 2.05^e^	151.47 ± 1.23^d^	97.45 ± 3.28^b^	38.00 ± 2.26^a^	256 ± 2^f^	0.52 ± 0.01^b^

FIC is expressed in fluorescence intensity (FI) per mg of sample DM. Browning is expressed as absorbance units (AU). Values are means ± standard deviation (*n *=* *3). Values in each column with different small superscript letters are significantly different (Fisher test, *P *≤* *0.05). DM, dry matter.

### Maillard reaction products evaluation

As shown in Table 4, furosine content decreased after spices addition from 4 up to 78% in comparison to cake without spices addition. The furosine contents in rye‐buckwheat ginger cakes after spices addition were significantly lower than the maximum allowable tolerance of furosine in milk proposed by Martysiak‐Żurowska and Stołyhwo (2007). Moreover the furosine content is even twice lower than determined in commercial breakfast cereals (Rada‐Mendoza et al. 2004) and five times lower in cookies made of wheat flour (Gökmen et al. 2008). The observation of inhibition effect of spices on furosine formation in cakes with spices was confirmed by high correlation between furosine and rutin (*r* = −0.80) as well as AC measured by ABTS (*r* = −0.81) and PCL ACW (*r* = −0.84). Whereas the weaker correlation (*r* = −0.68) between furosine and TPC contents was calculated. The strong relationship between furosine and OPA values and melanoidins formation were observed. The negative correlation coefficients were calculated (*r* = −0.91, *r* = −0.90, respectively), that can suggest that available lysine is a dominant precursor of MR progress in early stage and furosine formation is competitive to melanoidins. Whereas the positive correlation between furosine and FAST index data was noted (*r* = 0.81). It can be said that, in a great percent of furosine is converted into fluorescent compounds.

Collected in Table 4, the total, free, and linked‐to‐protein FIC found in all cakes were within the range of 142.4–201.7, 54.0–116.1, and 73.1–113.5 FI/mg sample DM, respectively. The total FIC values significantly decreased after cloves, spice mix, and allspice supplementation (15%, 10%, and 8%, respectively), the same effect was observed for free FIC data. It can be said that some spices promote fluorescence compounds formation, however between total FIC and rutin negative correlation was noted (*r* = −0.66). Between total FIC and OPA data according to calculated correlation coefficient (*r* = −0.72).

To describe protein nutritional loss, FAST index was calculated as a ratio between linked‐to‐protein fluorescence/tryptophan fluorescence and expressed in percentage. The strong correlation between tryptophan and TPC data and antioxidant ability measured by PCL ACW and ABTS assays (*r* = 0.92, 0.87, and 0.93, respectively) were noted. According to tryptophan results, it can be said that compounds from RBC supplemented with spices with strong antioxidant ability, have a potential to increase nutritional value. Table 4 shows the FAST index values. The values ranged from 230% to 484%. FAST index cloves, spice mix, cinnamon, nutmeg, and allspice being 2.0, 1.92, 1.8, 1.2, and 1.0 times lower. The positive influence of spices on FAST index decreasing was proved by correlation coefficient calculated between FAST/TPC, FAST/PCL, and FAST/ABTS (*r* = −0.80, *r* = −0.89, *r* = −0.81, respectively). According to results presented by Zieliński et al. (2012), rye ginger cakes showed a higher FAST index values in comparison to rye‐buckwheat ginger cakes. However the FAST index for rye ginger cakes before storage is twice lower than indexes for rye‐buckwheat ginger cakes. This indicates that measuring the FAST index in rye‐buckwheat ginger cakes before storage and then in a determined time intervals can be important to monitor ongoing changes.

Brown high molecular polymers of MRP pigments formation were determined and presented in Table 4. Addition of cloves, spice mix, allspice, cinnamon, nutmeg, and vanilla to the recipe significant increased (*P* < 0.05) browning index by 88%, 44% (both spice mix and allspice), 36%, 33%, and 14%, respectively. It has been proven that melanoidin formation is positively correlated with AC measured by ABTS test (*r* = 0.76) and PCL ACW assay (*r* = 0.65) and with TPC and rutin contents in the cakes (*r* = 0.63 and 0.86). These findings are in agreement with Zieliński et al. (2010), who also evaluated the positive correlation between the AC and melanoidin content in wheat‐rye ginger cakes. Moreover, browning index and OPA data were positively correlated (*r* = 0.89). The correlation value between FAST and browning indexes suggests that there is no relationship between loss of nutritional and melanoidin formation (*r* = ‐0.81).

## Conclusions

Among collection of spices, the RBC fortified individually with cloves, nutmeg, allspice, cinnamon, vanilla, and spice mix addition revealed the highest sensory characteristics and overall quality. Cakes fortified with cloves, allspice, and spice mix showed the highest AC, total phenolics, rutin, and almost threefold higher available lysine contents. The reduction in furosine content as well as free and total fluorescent intermediatory compounds was observed as compared to control nonfortified cakes. In contrast, browning index was increased as compared to cakes without spices. In this study, the chemical and sensorial markers were fully applicable for description of the quality of RBC fortified with spices. It can be suggested that cloves, allspice, vanilla, and spice mix should be used for production of safety and good quality cakes.

## Conflict of Interests

The authors declare that there is no conflict of interests regarding the publication of this paper.
